# Videoconferencing for multidisciplinary team meetings in the coronavirus disease era – human factors awareness and recognition

**DOI:** 10.1017/S0022215120002376

**Published:** 2020-11-04

**Authors:** C Kerawala, F Riva, V Paleri

**Affiliations:** 1 Head and Neck Unit, Royal Marsden NHS Foundation Trust, London, UK; 2 British Association of Head and Neck Oncologists, London, UK; 3 Faculty of Health and Wellbeing, University of Winchester, London, UK; 4 Institute of Cancer Research, London, UK

**Keywords:** COVID-19, Multi-Disciplinary Team, Patient Safety

## Abstract

**Background:**

The coronavirus disease 2019 pandemic has led to the birth of videoconference multidisciplinary teams, which are now commonplace. This remote way of deciding care demands a new set of rules to ensure the quality of the complex decisions that are made for the patient group needing multidisciplinary care. Videoconference multidisciplinary teams bring with them novel forms of distraction that are under-appreciated and can impair decision-making.

**Method:**

A practical checklist was generated as applied to videoconference multidisciplinary teams using the principles of human factors awareness and recognition.

**Results:**

Some of the strategies that should be adopted to minimise errors arising from human factors are: information technology support, a suitable environment to dial in, a global checklist employed prior to the videoconference, visible participants, avoiding distractions from other sources (e.g. e-mail, mobile phone), a videoconference sign-out and rapid dissemination of the outcomes sheet.

**Conclusion:**

This article presents a framework that uses human factors principles applied in this setting, which will contribute to enhanced patient safety, team working and a reduction in medical errors.

## Introduction

As a result of the coronavirus disease 2019 (Covid-19) pandemic, many institutions have reduced hospital attendance for clinicians and patients alike, in line with social distancing.^1,2^ The British Association of Head and Neck Oncologists was one of the first to suggest a reduction in the numbers of members that would constitute a quorate multidisciplinary team (MDT).^3,4^ Such recommendations saw the birth of videoconference MDTs, which are now commonplace.

This remote way of deciding care demands a new set of rules to ensure that the quality of the complex decisions which are made continue to fulfil the expectations of all involved. However, videoconference MDTs bring with them novel forms of distraction, and, as with other aspects of medical practice, the application of human factors awareness and recognition is timely.

Implementing safer practice using human factors principles contributes to enhanced patient safety, team working and a reduction in medical errors.^5,6^ When complex decisions are made during times of resource constraint, human factors assumes significant importance, both for individuals and the effective running of meetings. This article presents a framework that uses human factors principles to be applied in this setting, which will contribute to enhanced patient safety, team working and a reduction in medical errors.

## Discussion

Human failures can be categorised into four main domains: the influence of the employing organisation, preconditions to unsafe acts, unsafe supervision and unsafe acts themselves.^7^ The videoconference MDT exposes participants to many of the same potential failures as its face-to-face counterpart. Organisational influences such as a requirement to treat patients expediently still exist during the Covid-19 crisis.^8,9^ Errors due to unsafe acts and unsafe supervision should be no more prevalent given the continued presence of senior colleagues, who can mentor decisions and minimise skill-based or decision-making faults. However, as preconditions to unsafe acts often reflect circumstances personal to an individual, these have the potential to increase during videoconferencing, as decisions are made within an unfamiliar environment.

We are prone to distractions in everyday life, but the videoconference MDT brings with it new challenges and so a new set of rules (Table 1). Both external and internal distractions can be mitigated against, but only if their presence is both appreciated and pre-empted.

**Table 1.** Suggested videoconference MDT ‘rules’

Provide participants with a ‘users’ guide’ in case of IT problems
Arrange an IT ‘dummy run’
Ensure appropriate dial-in environment for participants
Commence with MDT checklist akin to WHO operating theatre checklist
Ensure participants are unmuted & visible throughout
Safeguard against distractions (e.g. smartphones)
Conclude MDT with ‘sign-out’
Ensure attendance sheet completed by MDT co-ordinator
E-mail outcomes sheet

MDT = multidisciplinary team; IT = information technology; WHO = World Health Organization

Participants should be provided with a user's guide to the information technology (IT) system in place, so that technical problems can be overcome. The participants should take part in a ‘dummy run’, such that confidence can be built up in the technology and teething problems ironed out in advance of a meeting where clinical decisions are made. Contributors should ensure that the environment from which they dial in is free from distractions and is appropriate given the confidential information that will be discussed. A specific videoconference MDT checklist should be employed at the beginning of each meeting to ensure that potential errors in process are minimised.

The hardware of the system employed should allow video and audio feeds of all participants in real time, so that the quorate nature of the meeting can be assessed throughout. Participants should not unmute or remove video images during the meeting – it is far too easy to lose concentration if one knows one is not being watched and as a result situational awareness may dwindle. It is likely that videoconference MDTs will become truncated as fewer patients will undergo intervention or be referred into secondary care giving clinicians less excuse to multitask. Smartphone use should not be permitted other than to videoconference because it is known to reduce performance.^10^ The chairperson should regularly ensure that participants are still able to hear and see all of the information being discussed; if any member is unable to hear or see any information, they should rapidly engage in a ‘hands-up’ signal – it is too easy to assume that lack of discussion reflects agreement rather than connectivity problems.

A checklist should be employed following each patient's discussion, such that the chair has the opportunity to ensure that the views of all participant members of the videoconference MDT have been recorded.

The videoconference MDT should be concluded with a ‘sign-out’ to ensure that everybody has been present throughout. The outcomes of the videoconference MDT discussions should be circulated by e-mail within 24 hours so that they can be ‘signed off’, as participants may not have seen real-time input into clinical records. An attendance sheet should be included.

Where an institution's IT support allows, adequate encryption consideration should be given to recording the meeting for future reference, as this could provide valuable evidence of collective decision-making in light of potential future challenges to treatment plans.

Many conventional face-to-face MDTs include a small social element such as a team coffee, as all too often colleagues talk about the loss of camaraderie in modern healthcare provision.^11,12^ A stable team engenders a supportive environment, which is needed more than ever. The very fact that clinicians are trying to avoid hospital footfall means that it is least available. A coffee break for five minutes at the beginning of the videoconference (at the end the coffee might be cold) might just be what the doctor ordered!

The above methodical approach to the increasingly commonplace videoconference MDT should facilitate participants’ engagement in the process and ensure robust discussions to the benefit of patient care.
